# Reduced Risk of Sepsis and Related Mortality in Chronic Kidney Disease Patients on Xanthine Oxidase Inhibitors: A National Cohort Study

**DOI:** 10.3389/fmed.2021.818132

**Published:** 2022-01-31

**Authors:** Huang-Yu Yang, Yun-Shiuan Olivia Hsu, Tao Han Lee, Chao-Yi Wu, Chung-Ying Tsai, Li-Fang Chou, Hui-Tzu Tu, Yu-Tung Huang, Shang-Hung Chang, Chieh-Li Yen, Meng-Hsuan Hsieh, Cheng-Chia Lee, George Kuo, Chih-Yen Hsiao, Hsing-Lin Lin, Jia-Jin Chen, Tzung-Hai Yen, Yung-Chang Chen, Ya-Chong Tian, Chih-Wei Yang, Gerard F. Anderson

**Affiliations:** ^1^Department of Health Policy and Management, Johns Hopkins Bloomberg School of Public Health, Baltimore, MD, United States; ^2^Nephrology Department, Kidney Research Institute, Chang Gung Memorial Hospital in Linkou, Chang Gung University College of Medicine, Taoyuan, Taiwan; ^3^Department of Medical Education, Chang Gung Memorial Hospital at Linkou, Taoyuan, Taiwan; ^4^Department of Dermatology, National Taiwan University Hospital, Taipei, Taiwan; ^5^Division of Allergy, Asthma, and Rheumatology, Department of Pediatrics, Chang Gung Memorial Hospital, Chang Gung University College of Medicine, Taoyuan, Taiwan; ^6^Center for Big Data Analytics and Statistics, Chang Gung Memorial Hospital, Linkou, Taiwan; ^7^Cardiovascular Department, Chang Gung Memorial Hospital at Linkou, Chang Gung University School of Medicine, Taoyuan, Taiwan; ^8^Graduate Institute of Nursing, Chang Gung University of Science and Technology, Taoyuan, Taiwan; ^9^Division of Nephrology, Department of Internal Medicine, Taoyuan General Hospital, Ministry of Health and Welfare, Taoyuan, Taiwan; ^10^Division of Nephrology, Department of Internal Medicine, Ditmanson Medical Foundation Chia-Yi Christian Hospital, Chiayi, Taiwan; ^11^Division of Critical Care Surgery, Department of Critical Care Medicine, Veterans General Hospital, Kaohsiung, Taiwan

**Keywords:** chronic kidney disease, sepsis, MACCE, febuxostat, allopurinol

## Abstract

**Background:**

Advanced chronic kidney disease (CKD) patients are at higher risk of sepsis-related mortality following infection and bacteremia. Interestingly, the urate-lowering febuxostat and allopurinol, both xanthine oxidase inhibitors (XOis), have been suggested to influence the sepsis course in animal studies. In this study, we aim to investigate the relationship between XOis and infection/sepsis risk in pre-dialysis population.

**Methods:**

Pre-dialysis stage 5 CKD patients with gout were identified through the National Health Insurance Research Database (NHIRD) in Taiwan from 2012 to 2016. Outcomes were also compared with national data.

**Results:**

In our nationwide, population-based cohort study, 12,786 eligible pre-dialysis stage 5 CKD patients were enrolled. Compared to non-users, febuxostat users and allopurinol users were associated with reduced sepsis/infection risk [hazard ratio (HR), 0.93; 95% confidence interval (CI), 0.87–0.99; *P* = 0.0324 vs. HR, 0.92; 95% CI, 0.86–0.99; *P* = 0.0163]. Significant sepsis/infection-related mortality risk reduction was associated with febuxostat use (HR, 0.68; 95% CI, 0.52–0.87). Subgroup analysis demonstrated preference of febuxostat over allopurinol in sepsis/infection-related mortality among patients younger than 65 years of age, stain users, non-steroidal anti-inflammatory drug non-users, and non-diabetics. There was no significant difference in major adverse cardiac and cerebrovascular event (MACCE) risk between users and non-users while reduced risk of all-cause mortality was observed for XOi users.

**Conclusions:**

Use of XOi in pre-dialysis stage 5 CKD patients may be associated with reduced risk of sepsis/infection and their related mortality without increased MACCE and overall mortality.

## Introduction

Preventive measures against infection in chronic kidney disease (CKD) patients are vital, since infectious complications are significant sources of both morbidity and mortality among these patients (1). One such complication is sepsis, which is defined as life-threatening organ dysfunction caused by a dysregulated host response to infection (2). Interestingly, uric acid (UA), the end product of purine metabolism that is often elevated in CKD patients, has also been suggested as a biomarker for sepsis severity (3), while serum UA level at the initiation of dialysis has been associated with infection-related mortality (4). Furthermore, gout has been associated with increased risk of cancer while hyperuricemia predicts increased cancer mortality risk (5). Taken together the observed connection of gout with both infection and cancer, it is reasonable to hypothesize that gout patients may suffer an impairment or dysfunction of the immune system.

Not surprisingly, marked increase in the activity of xanthine oxidase, an enzyme necessary in the generation of uric acid, is also observed in non-surviving septic patients (6). Therefore, targeting xanthine oxidase and thus uric acid production may potentially influence infection and sepsis risk. Allopurinol and febuxostat are the two commonly prescribed xanthine oxidase inhibitors (XOis) used for the treatment of gout in CKD. In studies of septic mice and rats, allopurinol treatment has demonstrated both protection against sepsis and aggravation of sepsis (7). Mice treated with febuxostat has exhibited reduced sepsis-induced liver and kidney injuries and mortality (8, 9). In humans, one clinical trial has actually reported upper respiratory tract infection as a frequently occurring adverse effect for both allopurinol users and febuxostat users (10). Since studies on the role of XOis in infection/sepsis risk have been largely animal-based and scarce, no conclusion can be drawn at present.

In our nationwide population-based cohort study, we aimed to investigate the relationship between xanthine oxidase inhibitor use (either allopurinol or febuxostat) and the risk of infection/sepsis and their related mortality in CKD patients. Since lower estimated glomerular filtration rate (eGFR) in CKD has been correlated with higher risk of infection-related death (11), we exclusively selected stage 5 CKD patients, those predicted as most vulnerable to infectious complications, in this study. The commonly debated concerns of XOi, i.e., major adverse cardiac and cerebrovascular event (MACCEs) and all-cause mortality, were also evaluated.

## Methods

### Data Source

The present study used data from the NHIRD, which contained complete healthcare utilization data of ~24 million persons enrolled under the universal National Health Insurance (NHI) program in Taiwan and has been demonstrated to be a reliable source for population studies (12). This study complied with the Declaration of Helsinki and Declaration of Taipei (on ethical considerations regarding health databases and biobanks) of the World Medical Association and was approved (approval serial number: 201801872B0) by the institutional review board at Chang Gung Medical Foundation. Informed consent was waived due to using administrative data with de-identified (encrypted) personal information.

### Design and Study Participants

This study was a population-based retrospective cohort study. We selected patients who had at least twice been diagnosed with both CKD and gout. The diagnostic codes from the *International Classification of Diseases, Ninth Revision, Clinical Modification* (ICD-9-CM) and ICD-10 were used to define the diseases (Supplementary Table 1). Erythropoietin-stimulating agent (ESA) prescription was utilized to ascertain stage 5 CKD status, since the NHI program reimbursed its use in CKD patients with serum creatinine >6 mg/dL and hematocrit ≤28% before November 30, 2015. These reimbursement criteria guaranteed stage 5 CKD status (eGFR <15 mL/min/1.73 m^2^) based on the 4-variable Modification of Diet in Renal Disease Study equation and the Chronic Kidney Disease Epidemiology Collaboration (CKD-EPI) equation (13, 14). Starting on December 1, 2015, the NHI altered the reimbursement criteria to stage 5 CKD patients with hemoglobin level <9 g/dL. Thus, patients who satisfied the above criteria with a first ESA prescription date between January 1, 2012 and December 31, 2016, were initially selected for this study.

**Figure 1:**
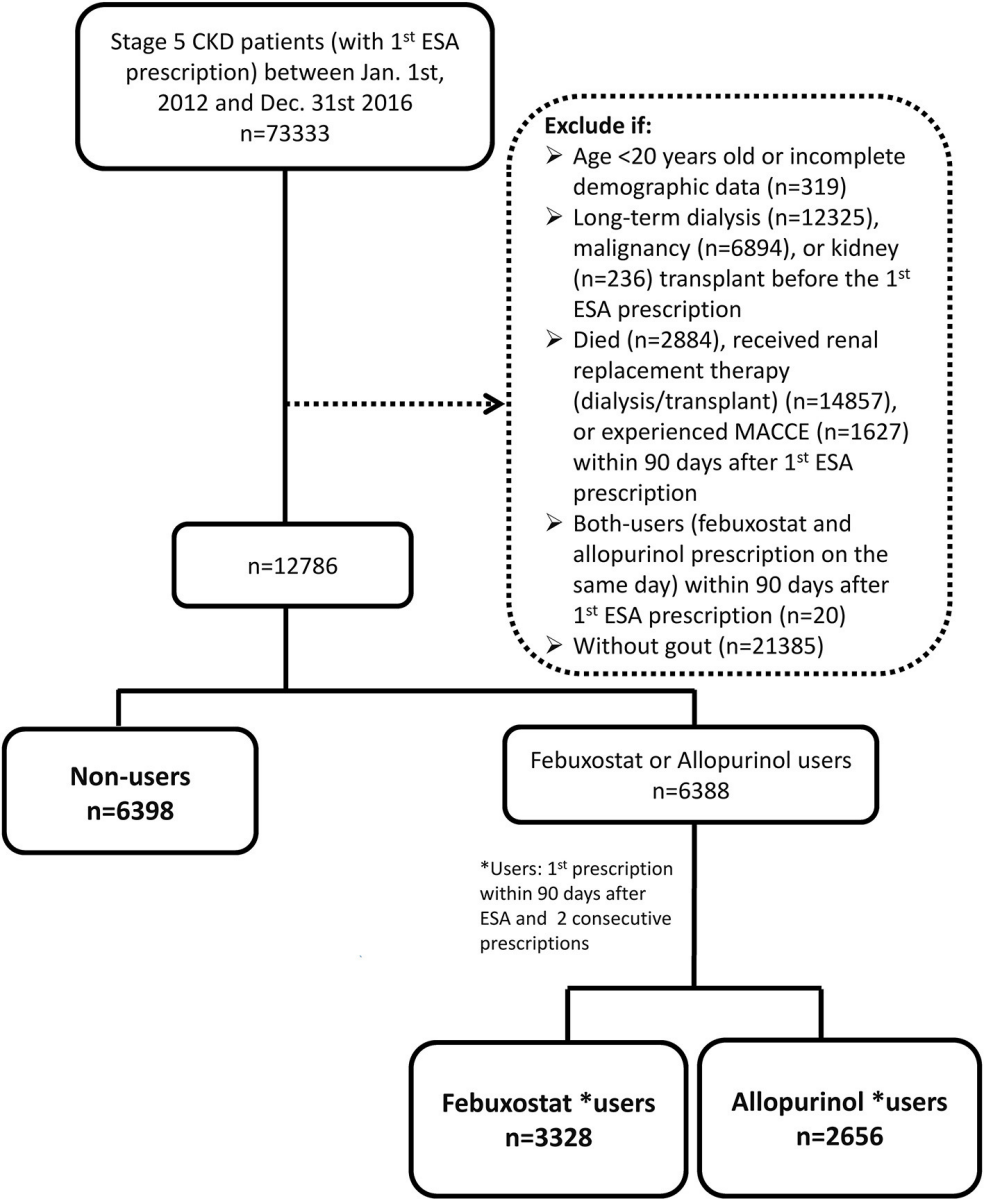
Flowchart of patient selection.

We excluded patients with incomplete demographic data, those younger than 20 years (legal age based on the Civil Code), and those who had received renal replacement therapy before ESA prescription. Patients who had been diagnosed with cancer prior to ESA prescription since ESA was reimbursed for cancer chemotherapy-related anemia. Since we used prescription information within 90 days after ESA treatment to ascertain anti-hyperuricemic use, the 91st day after ESA prescription was set as the index date (15). Patients who died, required renal replacement therapy, or experienced MACCEs within 90 days after ESA prescription were also excluded (Figure 1). We were able to control for survival bias by selecting patients who survived to the 91st day after ESA prescription and following them after this exposure time window (16).

Patients with febuxostat or allopurinol prescription within 90 days after ESA prescription and at least one consecutive prescription of the same drug thereafter were categorized as febuxostat users or allopurinol users. Non-users were patients without prescription of either XOi within 90 days after ESA prescription. All analyses were conducted on an intention-to-treat basis. Other covariates, such as comorbidities, were defined as diseases with at least two outpatient diagnoses or one inpatient diagnosis within 3 years before the index date. Medication use was defined as having any prescription record after the index date.

### Outcomes: Sepsis/Infection, Sepsis/Infection-Related Mortality, Major Adverse Cardiac and Cerebrovascular Events, and All-Cause Mortality

The observation period started on the 91st day after ESA prescription (the index date) and ended on the date of death or December 31, 2017 (the end date of the study), whichever came first. The outcomes of interest were sepsis/infection, sepsis/infection-related mortality, MACCEs, and all-cause mortality. The diseases included in MACCEs and sepsis/infection in this study are listed in Supplementary Table 1. The outcomes were identified by having the corresponding primary diagnosis as inpatients or emergency department visits (Supplementary Table 1) or the underlying cause of death.

### Statistical Analysis

For propensity score weighting (PSW), we used stabilized weights to preserve the sample size of the original data, produce appropriate estimation of the variance of main effect, and maintain an appropriate type I error rate (17). Absolute standardized mean difference (ASMD) was applied to compare baseline characteristics between propensity-score weighted groups, with ASMD ≤0.1 indicating non-significant difference. The Kaplan-Meier Estimator was applied to generate survival probability curves, which were compared by the log-rank test. We applied the multivariate Cox proportional hazards model, which adjusted for age, sex, place of residence, income level, occupation, comorbidities, and other medication use to compare the risks of the anticipated outcomes. The proportional hazards assumption was checked and met using the log–log plot. For the sepsis/infection and MACCE outcomes, observations were censored on the date of death or the end of the study. Subgroup analyses comparing febuxostat and allopurinol use were performed for the sepsis/infection and the sepsis/infection-related mortality outcomes. All *p-*values were two-sided, and the significance α level was set at 0.05. All statistical analyses were performed using SAS version 9.4 (SAS Institute).

## Results

### Patient Characteristics

The study initially included 12,786 pre-dialysis advanced CKD patients with gout. Non-users (*n* = 6,398) were patients without prescription of any XOi within 90 days after ESA prescription. Patients who were prescribed the same XOi at least twice consecutively were included and divided into febuxostat users (*n* = 3,328) and allopurinol users (*n* = 2,656). The mean age of patients was 71, 69, and 67 years among non-users, febuxostat users, and allopurinol users, respectively (Table 1). Except for the non-user group, which consisted of 49.17% male, both XOi user groups were predominantly male. The most common comorbidities among all groups were hypertension, diabetes mellitus, and dyslipidemia, in the order of most to least common. Supplementary Table 2 shows the patient characteristics after propensity score weighting, which demonstrates no significant difference across all variables with the exception of place of residence.

**Table 1:** Baseline Patient Characteristics before Propensity Score Weighting.

	**Non-users (*n* = 6,398)**	**Febuxostat users (*n* = 3,328)**	**Allopurinol users (*n* = 2,656)**	**ASMD**
Age, year, mean (SD)	71 (12)	69 (13)	67 (13)	0.2919
Male, no. (%)	3,146 (49.17)	2,175 (65.35)	1,830 (68.90)	0.4095
**Place of residence, no. (%)**				0.0463
Urban	3,368 (52.6)	1,755 (52.73)	1,433 (53.95)	
Suburban	2,168 (33.89)	1,120 (33.65)	852 (32.08)	
Rural	844 (13.19)	442 (13.28)	362 (13.63)	
Unknown	18 (0.28)	11 (0.33)	9 (0.34)	
**Income levels, no. (%)**				0.0711
Quintile 1 (Lowest)	1,683 (26.31)	819 (24.61)	594 (22.34)	
Quintile 2	1,355 (21.18)	288 (8.65)	744 (28.01)	
Quintile 3	1,423 (22.24)	1,153 (34.65)	403 (15.17)	
Quintile 4	716 (11.19)	384 (11.54)	351 (13.22)	
Quintile 5 (Highest)	1,219 (19.05)	683 (20.52)	564 (21.23)	
Unknown	2 (0.03)	1 (0.03)	–	
**Occupation, no. (%)**				0.1637
Dependents of the insured individuals	2,570 (40.17)	1,288 (38.70)	988 (37.20)	
Civil servants, teachers, military personnel and veterans	597 (9.33)	305 (9.16)	230 (8.66)	
Non-manual workers and professionals	403 (6.30)	274 (8.23)	264 (9.94)	
Manual workers	2,151 (33.62)	1,081 (32.48)	928 (34.94)	
Other	677 (10.58)	380 (11.42)	246 (9.26)	
**Comorbidities, no. (%)**
Diabetes mellitus	4,287 (67.01)	2,251 (67.64)	1,515 (57.04)	0.2200
Hypertension	6,124 (95.72)	3,182 (95.61)	2,553 (96.12)	0.0256
Dyslipidemia	3,771 (58.94)	2,014 (60.52)	1,513 (56.97)	0.0722
Liver cirrhosis	272 (4.25)	159 (4.78)	119 (4.48)	0.0254
SLE	53 (0.83)	32 (0.96)	15 (0.56)	0.0456
Atrial fibrillation	403 (6.30)	216 (6.49)	147 (5.53)	0.0402
Peripheral arterial disease	455 (7.11)	189 (5.68)	131 (4.93)	0.0917
**Medications use, no. (%)**
ACEi	419 (6.55)	157 (4.72)	184 (6.93)	0.0945
ARB	2,552 (39.89)	1,430 (42.97)	1,142 (43.00)	0.0632
CCB	4,753 (74.29)	2,588 (77.76)	2,096 (78.92)	0.1095
Beta-blockers	2,061 (32.21)	1,128 (33.89)	948 (35.69)	0.0735
Diuretics	3,962 (61.93)	2,084 (62.62)	1,613 (60.73)	0.0389
Aspirin	1,391 (21.74)	730 (21.94)	644 (24.25)	0.0596
Other NSAIDs	1,798 (28.10)	989 (29.72)	840 (31.63)	0.0771
Fibrates	258 (4.03)	162 (4.87)	140 (5.27)	0.0588
Statin	2,015 (31.49)	1,304 (39.18)	906 (34.11)	0.1614
Thiazolidinedione	133 (2.08)	72 (2.16)	54 (2.03)	0.0091

*ACEi, angiotensin converting enzyme inhibitor; ARB, angiotensin II receptor blocker; ASMD, absolute standardized mean difference; CCB, calcium channel blocker; NSAIDs, non-steroidal anti-inflammatory drugs; SLE, systemic lupus erythematosus*.

### Outcomes: Sepsis/Infection and Sepsis/Infection-Related Mortality, MACCE, and All-Cause Mortality

Four outcomes were examined during the period from 90 days after January 1, 2012 to December 31, 2017 in pre-dialysis stage 5 CKD patients. These outcomes included sepsis/infection, sepsis/infection-related death, MACCEs, and all-cause mortality. Outcome definitions were stated in *Methods*.

**Figure 2:**
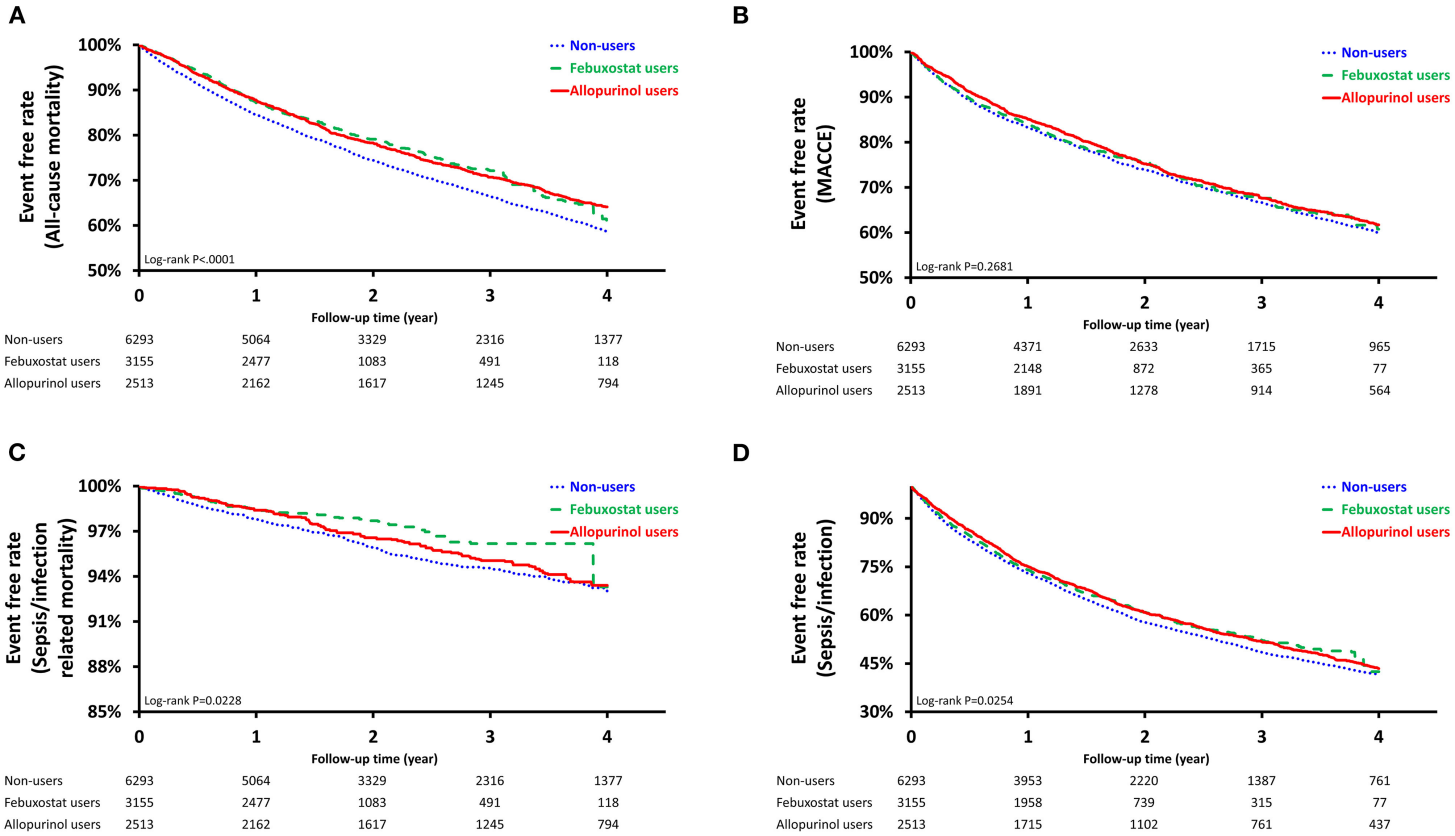
Survival Probability of Pre-Dialysis Stage 5 CKD Patients. Survival curves were created using the Kaplan Meier estimator. **(A)**: Sepsis/Infection (Log-rank test, *P* < 0.0254); **(B)**: Sepsis/Infection-Related Mortality (Log-rank test, *P* < 0.0228); **(C)**: Major Adverse Cardiac and Cerebrovascular Events, MACCEs (Log-rank test, *P* < 0.2681); **(D)**: All-Cause Mortality (Log-rank test, *P* < 0.0001).

For the sepsis/infection outcome, the incidence was highest among non-users of XOis (25.67 per 100 person-years; Table 2). Compared to the reference group (non-user group), both febuxostat users and allopurinol users had lower risk of sepsis or infection [hazard ratio (HR), 0.93; 95% confidence interval (CI), 0.87–0.99; *P* = 0.0324 vs. HR, 0.92; 95% CI, 0.86–0.99; *P* = 0.0163]. Survival curves by means of the Kaplan-Meier estimator were generated and analyzed using the log-rank test. Sepsis/infection survival curves similarly demonstrated significant survival benefit for febuxostat and allopurinol users (Figure 2A).

**Table 2:** Outcome incidences and hazard ratios.

	**Before PSW**			**After PSW**		
	**No. of event/person-years**	**Incidence rate (95% CI) per 100 person-years**	**Hazard ratio (95% CI); *P*-value**	**No. of event/person-years**	**Incidence rate (95% CI) per 100 person-years**	**Hazard ratio (95% CI); *P*-value**
**Sepsis/infection**						
Non-users	2,893/1,0797.87	26.79 (25.82–27.77)	Reference	2,787/1,0858.89	25.67 (24.71–26.62)	Reference
Febuxostat users	1,166/4,669.9	24.97 (23.54–26.40)	0.87 (0.81–0.93); *P* < 0.0001	1,148/4,535.07	25.31 (23.84–26.77)	0.93 (0.87–0.99); *P* = 0.0324
Allopurinol users	1,192/5,657.95	21.07 (19.87–22.26)	0.82 (0.77–0.88); *P* < 0.0001	1,146/4,992.15	22.96 (21.63–24.29)	0.92 (0.86–0.99); *P* = 0.0163
**Sepsis/infection-related mortality**						
Non-users	295/1,4551.41	2.03 (1.8–2.26)	Reference	280/1,4515.8	1.93 (1.7–2.15)	Reference
Febuxostat users	76/5,844.7	1.3 (1.01–1.59)	0.62 (0.48–0.8); *P* = 0.0002	76/5,708.15	1.33 (1.03–1.63)	0.68 (0.52–0.87); *P* = 0.0027
Allopurinol users	107/7,490.06	1.43 (1.16–1.7)	0.72 (0.58–0.9); *P* = 0.0034	113/6,665.77	1.69 (1.38–2.01)	0.89 (0.71–1.10); *P* = 0.2834
**MACCE**						
Non-users	1,794/12,200.04	14.70 (14.02–15.39)	Reference	1,765/1,2177.18	14.50 (13.82–15.17)	Reference
Febuxostat users	775/5,048.18	15.35 (14.27–16.43)	0.97 (0.89–1.06); *P* = 0.5331	738/4,937.29	14.95 (13.87–16.02)	0.97 (0.89–1.06); *P* = 0.4719
Allopurinol users	775/6,278.94	12.34 (11.47–13.21)	0.88 (0.81–0.95); *P* = 0.0020	732/5,574.63	13.14 (12.19–14.09)	0.93 (0.85–1.01); *P* = 0.0963
**All-cause mortality**						
Non-users	2,168/1,4551.41	14.90 (14.27–15.53)	Reference	2,041/1,4515.8	14.06 (13.45–14.67)	Reference
Febuxostat users	672/5,844.7	11.50 (10.63–12.37)	0.74 (0.68–0.81); *P* < 0.0001	687/5,708.15	12.03 (11.13–12.93)	0.82 (0.76–0.90); *P* < 0.0001
Allopurinol users	771/7,490.06	10.29 (9.57–11.02)	0.71 (0.65–0.77); *P* < 0.0001	768/6,665.77	11.53 (10.71–12.34)	0.83 (0.77–0.90); *P* < 0.0001

*PSW, Propensity score weighting*.

The sepsis/infection-related mortality incidence was lowest among febuxostat users both before and after PSW (1.3 per 100 person-years vs. 1.33 per 100 person-years; Table 2). Though both febuxostat users and allopurinol users had lower risk of sepsis or infection-related mortality, only the former exhibited significant risk reduction after PSW (HR, 0.68; 95% CI, 0.52–0.87; *P* = 0.0027 vs. HR, 0.89; 95% CI, 0.71–1.10; *P* = 0.2834). Significant differences in survival among non-users, febuxostat users, and allopurinol users were similarly demonstrated after propensity-score weighting (*P* = 0.0228; Figure 2B).

Before PSW, allopurinol users were associated with the lowest incidence of MACCEs (12.34 per 100 person-years; Table 2) and lower risk of MACCE compared to non-users (HR, 0.88; 95% CI, 0.81–0.95; *P* = 0.0020). No significant difference was observed in MACCE-free survival among the three groups after PSW (*P* = 0.2681; Figure 2C). Both febuxostat use and allopurinol use were associated with reduced risk of all-cause-mortality after PSW (HR, 0.82; 95% CI, 0.76–0.90; *P* < 0.0001 vs. HR, 0.83; 95% CI, 0.77–0.90; *P* < 0.0001; Table 2). After PSW, significant difference in survival among the three groups was observed (*P* < 0.0001; Figure 2D).

### Subgroup Analysis: Sepsis/Infection and Sepsis/Infection-Related Mortality

**Figure 3:**
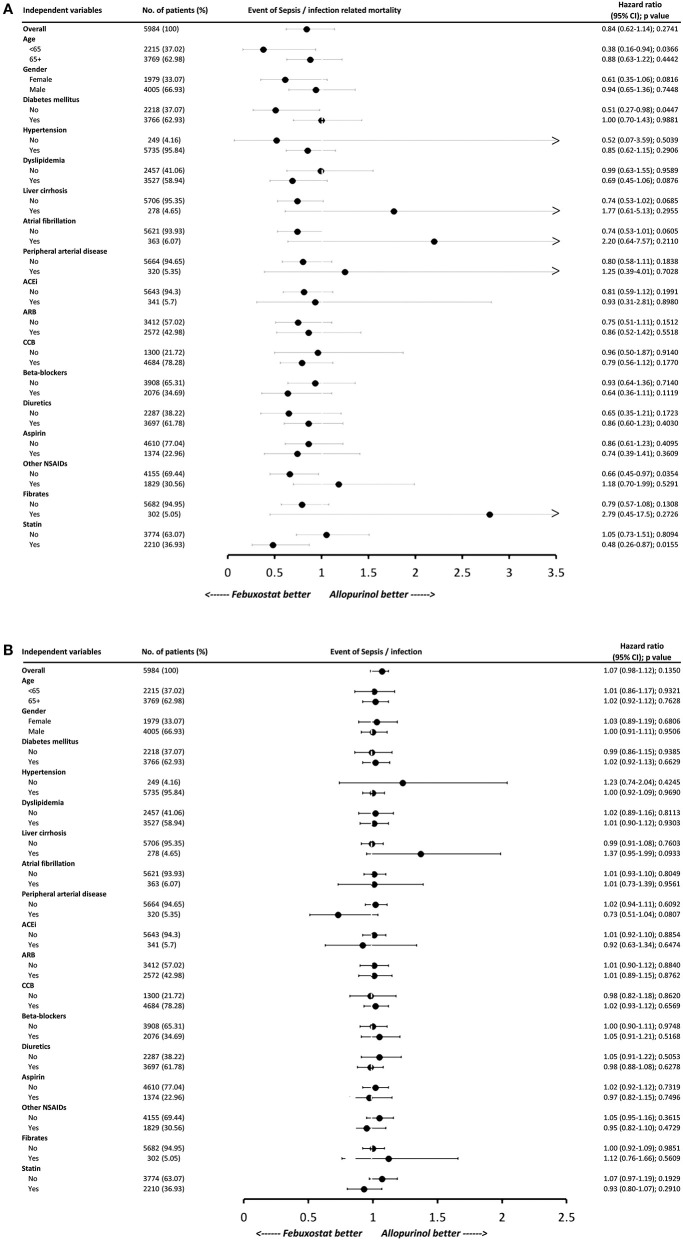
Subgroup analysis of **(A)** sepsis/infection-related mortality and **(B)** sepsis/infection. Hazard ratios (HRs) less than one favors febuxostat use. Each variable was adjusted for all other variables listed in Table 1. HBV indicates hepatitis B virus infection; HCV, hepatitis C virus infection; SLE, systemic lupus erythematosus; ACEi, angiotensin converting enzyme inhibitor; ARB, angiotensin II receptor blocker; CCB, calcium channel blocker; NSAID, non-steroidal anti-inflammatory drug.

To determine whether patients with specific comorbid conditions were associated with reduced risk of sepsis/infection and their related mortality when choosing one XOi over the other, subgroup analysis was performed (Figures 3A,B). Comparison between allopurinol and febuxostat showed no significant difference in the risk of developing sepsis or infection in all subgroups. On the other hand, for sepsis/infection-related mortality, febuxostat use was associated with significant risk reduction for those younger than 65 years of age (HR, 0.38; 95% CI, 0.16–0.94; *P* = 0.0366), non-diabetics (HR, 0.51; 95% CI, 0.27–0.98; *P* = 0.0447), NSAID non-users (HR, 0.66; 95% CI, 0.45–0.97; *P* = 0.0354), and statin users (HR, 0.48; 95% CI, 0.26–0.87; *P* = 0.0155).

## Discussion

In our large, population-based cohort of pre-dialysis stage 5 CKD patients, xanthine oxidase inhibitor use was associated with significant reduction in risk of sepsis or infection. As for sepsis/infection-related mortality, though both XOis demonstrated risk reduction, only febuxostat showed significance. Both drugs were also associated with non-significantly decreased MACCE risk and significantly reduced all-cause mortality risk when compared to non-users. These findings suggest the overall safety of both XOis in advanced CKD.

Oxidative stress is implicated in the pathomechanism of systemic inflammatory response syndrome and sepsis-induced organ failure (18). Interestingly, uric acid has a complex role in oxidative stress as both a scavenger of free radicals (anti-oxidant) and a pro-oxidant (19). It has been previously demonstrated in a rabbit study that allopurinol, via its antioxidant effects, reduced atrial remodeling induced by diabetes mellitus-related increases in oxidative stress (20). In lipopolysaccharide-(LPS-)induced sepsis in mice, coadministration of allopurinol aggravated septic shock, leading to mortality, renal function impairment, high reactive oxygen species, and high proinflammatory interleukin levels while coadministration of febuxostat did not potentiate sepsis (7). However, studies of septic mice and rats have suggested both protective and aggravating effects of allopurinol on sepsis (7, 21). As for febuxostat, one mice study has exhibited reduced sepsis-induced liver and kidney injuries, oxidative stress, and mortality (9). Another study similarly demonstrated accelerated recovery of pulmonary endothelial barrier and improved survival in LPS-induced septic mice treated with febuxostat (8). Evidence from animal studies so far seemed to suggest febuxostat as neutral or beneficial, echoing our result that febuxostat users were associated with significant reduction of sepsis/infection-related mortality. An explanation for the non-significant risk reduction among allopurinol users may be the difficulty in achieving optimal allopurinol dose in CKD for adequate gout control (22). One study showed that patients with escalated allopurinol dose experienced an increase in all-cause mortality (23). Furthermore, underlying mechanistic differences between the two XOis may contribute to the difference but are beyond the scope of our cohort study. Whether observations from animal studies could be applied to humans still warrant investigation.

It is also possible that uric acid and xanthine oxidase inhibitors alter the immune response in the event of infection. In the context of viral infection in humans, significantly increased levels of uric acid were found in lung aspirate samples of hospitalized infants positive for RSV infection (24). Further investigation in mice demonstrated that uric acid upregulation during RSV infection exacerbated the Th2 response, while treatment with allopurinol demonstrated reduction of inflammatory infiltrates and mucus in the airways and Th2 cytokines. However, whether uric acid plays a role in the immune response against non-pulmonary or non-viral infections still warrants further investigation.

With the increase in XOi use among gouty CKD patients, potential side effects of these medications have become important. This rise in prescription is at least partially contributed to hyperuricemia's suggested role in the progression to end-stage renal disease with studies demonstrating renoprotective potential of XOis (25–28). Furthermore, hyperuricemia is widely accepted as a risk factor for cardiovascular diseases, perhaps partially due to promotion of chronic formation of neutrophil extracellular traps, thereby contributing to vascular damage (29). Ironically, cardiovascular (CV) event risk associated with XOi use has been an unresolved topic of debate. The U.S. Food and Drug Administration previously warned against increased CV risk of febuxostat based on comparison with allopurinol in a randomized controlled trial by White et al. (30). On the other hand, the FAST trial has demonstrated that febuxostat is non-inferior to allopurinol with respect to the primary cardiovascular events without an increased risk of death or serious adverse events, including infections (31), further suggesting febuxostat's relative safety. In fact, several cohort studies have found that XOi use (either allopurinol or febuxostat) is not associated with change in cardiovascular risk and even proposed reduced risk of CV events with allopurinol use (32, 33). As for patients with CKD, the FEATHER trial has found no increase in cardiovascular events with febuxostat in comparison to placebo in CKD stage 3 patients (34). In terms of overall survival, previous studies have suggested improvement with allopurinol use (35, 36). When comparing the all-cause mortality of the two XOis, febuxostat has been found as both comparable and at higher risk to allopurinol (37). However, in our previous study, febuxostat was associated with superior renoprotection without compromising survival compared to allopurinol in CKD 5 patients (38). Without a consensus, further studies focusing on the mortality risk of the different XOis in CKD 5 patients are necessary.

In the present national cohort study, the gender distribution is not matched with the control group, as both XOi user groups were predominantly male. It is possible that the different baseline gender distribution between groups does not reflect disease severity, but rather, merely differential clinician practice. Studies have shown that the prescription rate of medications may be inherently different between genders. In a United Kingdom study of 40 practices with 300,000 people, compared with men with gout, prescription of allopurinol was lower for women with gout across all age strata, except for the highest age group (39). Second, it is also plausible to infer that the non-user group is not treated with anti-hyperuricemic agents because these patients are diagnosed with gout more recently. It has been demonstrated that as compared with men with gout, women with gout were older (mean age 70 vs. 58, *p* < 0.001) (40), consistent with the finding that menopause increases the risk of gout and that postmenopausal hormone therapy modestly reduces gout risk (41). Therefore, it is reasonable that the slightly female-predominant non-user group consisted of more newly diagnosed gout patients, hence no febuxostat or allopurinol use yet. If this inference were true, whether these patients were associated with lower risks of the study outcomes deserved future investigation and would provide further evidence that treatment with anti-hyperurecemic agents are associated with reduced risk of our study outcomes. Third, we should consider whether gender solely could modulate the risks of study outcomes, especially those that demonstrated statistical significance in our study. Interestingly, data from a recent Coronavirus disease 2019 study has found higher mortality among men and suggested the protective role of estradiol (42). Advanced CKD patients on dialysis often suffer from genitourinary, pulmonary, septicemia, and CVC related infections, with women having higher infection rates (43). However, both genders have been reported to have higher mortality rate following severe infection or sepsis (44–47). Therefore, whether gender should be considered a reliable predictor of infection-related mortality requires further validation. In terms of all-cause mortality among CKD patients, though men are observed to have higher all-cause mortality than women at all levels of eGFR (48), our male-dominant febuxostat or allopurinol group is still associated with mild but significant reduction in all-cause mortality compared to the non-user group. Last but not least, the data presented in Table 2 were propensity-scored weighted and adjusted for possible confounding factors listed in Table 1, including gender.

Our subgroup analyses found that younger patients (<65 years of age), non-diabetics, NSAID non-users, and statin users prescribed febuxostat were associated with significant decrease in the risk of sepsis/infection-related death compared to those prescribed allopurinol. It is possible that having fewer comorbidities contributed to the reduced risk of sepsis/infection-related mortality in younger patients using febuxostat. Since studies comparing the sepsis risk of the two medication are lacking, further postulations are difficult to make based on our cohort study. In our study, febuxostat was preferred over allopurinol in non-diabetics in term of decreased risk of sepsis/infection-related death. One possible explanation is their difference in urate lowering efficacy. Becker et al. previously reported that patients using febuxostat (40 mg/day) with moderate renal impairment showed lower efficacy in diabetic than in non-diabetic gout patients, demonstrating that diabetics required higher dose to achieve comparable urate lowering effect (49). However, they observed that allopurinol had lower urate lowering efficacy compared to febuxostat regardless of diabetes status. Furthermore, diabetics and non-diabetics had similar rates of upper respiratory infection, though it is not known if a certain medication contributed more to this observation. Interestingly, studies on febuxostat and statins have largely focused on myopathy risk. While a few studies have suggested that CKD with concomitant statin or fibrate therapy may represent increased risk for febuxostat-induced rhabdomyolysis (50), Liu et al. have found that patients with severely reduced eGFR had higher risk of febuxostat-associated myopathy with or without concurrent statin or fibrate use (51). On the other hand, febuxostat but not allopurinol was recently suggested to increase the oral bioavailability of rosuvastatin. How this newly discovered drug-drug interaction contributes to the overall CV risk and other complications, such as sepsis/infection risk, is at a nascent stage requiring further investigation.

To our knowledge, this study is the first large, real-world cohort study to evaluate the sepsis and infection risk in pre-dialysis stage 5 CKD patients prescribed xanthine oxidase inhibitors. We have demonstrated that both allopurinol and febuxostat are associated with reduced risk of sepsis/infection and their related mortality without increasing the risk of MACCE or all-cause mortality. Nonetheless, several limitations are present in this study. First of all, due to the limited information provided by the NHIRD, we could not correlate our observed outcomes with changes in biochemical data, such as levels of uric acid, cytokines, or other useful markers. Second, the dosage of allopurinol and febuxostat was unknown and unable to be controlled. Third, since our study was retrospective and observational in design, no causal relationship or mechanism could be proved. Ideally, randomized controlled trials (RCTs) should be performed for more definitive conclusion.

## Conclusion

The use of febuxostat and allopurinol appears to associate with decreased risk of sepsis and infection and their related death in pre-dialysis stage 5 CKD patients without compromising CV health and overall survival. Future RCTs or mechanistic studies are warranted to confirm and elucidate the comprehensive functions of xanthine oxidase inhibitors, especially in patients with compromised renal function.

## Data Availability Statement

The original contributions presented in the study are included in the article/Supplementary Material, further inquiries can be directed to the corresponding author/s.

## Ethics Statement

The studies involving human participants were reviewed and approved by Institutional Review Board at Chang Gung Medical Foundation. Written informed consent for participation was not required for this study in accordance with the national legislation and the institutional requirements.

## Author Contributions

Y-SH, C-YW, and H-YY wrote the manuscript. Y-SH, C-YT, L-FC, S-HC, C-LY, M-HH, C-CL, THL, and H-YY designed the research. H-TT, THL, J-JC, and Y-TH performed the research. Y-SH, C-YW, GK, C-YH, H-LL, T-HY, Y-CC, Y-CT, C-WY, GA, and H-YY analyzed the data. H-TT and Y-TH contributed new reagents/analytical tools. All authors contributed to the article and approved the submitted version.

## Funding

This study was supported by grants from Chang Gung Memorial Hospital, Taiwan (CMRPG3G1233 and CORPG3J0641).

## Conflict of Interest

The authors declare that the research was conducted in the absence of any commercial or financial relationships that could be construed as a potential conflict of interest.

## Publisher's Note

All claims expressed in this article are solely those of the authors and do not necessarily represent those of their affiliated organizations, or those of the publisher, the editors and the reviewers. Any product that may be evaluated in this article, or claim that may be made by its manufacturer, is not guaranteed or endorsed by the publisher.
