# Supplementary material for: Reduced Risk of Sepsis and Related Mortality in Chronic Kidney Disease Patients on Xanthine Oxidase Inhibitors: A National Cohort Study
Source: Front Med (Lausanne). 2022 Jan 31;8:818132. doi: 10.3389/fmed.2021.818132 (PMC8841527; doi:10.3389/fmed.2021.818132)
Supplement: Supplementary file 1 [file Data_Sheet_1.docx]

**SUPPLEMENTARY MATERIALS**

**Table S1.** List of ICD-9-CM, ICD-10, and Procedure Codes

**Table S2.** Baseline Characteristics of Patients *after* Propensity-Score Weighting

**Table S1.** List of ICD-9-CM, ICD-10, and Procedure Codes

| **Diagnosis** | **ICD-9-CM/ Procedure Code** | **ICD-10/ Procedure Code** |
| --- | --- | --- |
| CKD | 016.0, 042, 095.4, 189, 223, 236.9, 250.4, 271.4, 274.1, 403-404, 440.1, 442.1, 446.21, 447.3, 572.4, 580-589, 590-591, 593, 642.1, 646.2, 753, 984 | A18.11, B20, A52.75, C64.9, C65.9, C66.9, C68.0, C68.1, C68.8, C68.9, D30, D41, E11.2, E74.8, M10.30, N20.0, I12, I13, I70.1, I72.2, M31.0, I77.3, K76.7, N00-N19, N25-N29, O10.4, O26.83, O90.89, Q60-Q64, T56.0 |
| Gout | 274 | M10 |
| DM | 250 | E8-E11, E13 |
| Hypertension | 401, 402, 403, 404, 405 | I10-I13, I15-I16 |
| dyslipidemia | 272 | E78 |
| Liver cirrhosis | 571.2, 571.5, 571.6 | K70.3, K74.3-K74.6 |
| SLE | 710.0 | M32 |
| Atrial fibrillation | 427.31 | I48.91 |
| Peripheral vascular disease | 443 | I73 |
| **Major Adverse Cardiac and Cerebrovascular Event (MACCE)** |  |  |
| Cardiogenic shock | 785.51 | R57.0 |
| Heart failure | 428 | I50 |
| Malignant dysrhythmia | 426.0, 426.12-426.13, 426.51-426.52, 426.54, 427.1, 427.4, 427.41-427.42, 427.5 | I44.0-3, I45.2, I45.3, I46.9, I47.2, I49.0-3 |
| Myocardial infarction | 410 | I21, I22 |
| Stroke | 430-437 | I60-64, G45.0, G45.1, G45.4, G45.8, I67 |
| Percutaneous Transluminal Coronary (PCI) | 33076A, 33076B, 33077A, 33077B, 33078A, 33078B | 02103,02104, 02113, 02114, 02123, 02124, 02133, 02134 |
| Coronary artery bypass surgery (CABG) | 68023A, 68023B, 68024A, 68024B, 68025A, 68025B | 02100, 02110,02120, 02130 |
| Thrombolysis therapy (TT) | B016526248, K000743248, K000744238 | 3E03317 |
| **Infectious disease** |  |  |
| Sepsis | 038, 995.91, 995.92, 020.2, 785.52, 790.7 | A40, A41, R65.20, A20.7, R65.21, R78.81 |
| Pneumonia | 481-486 (exclude 484) | J13-J18 (exclude J17) |
| Empyema | 510 | J86.0, J86.9 |
| Cellulitis | 681, 682 | L03 |
| Necrotizing fasciitis | 728.86 | M72.6 |
| Urinary tract infection | 590, 595.0, 599.0 | N10-N12, N15.1, N15.9, N16, N28.85, N30.00, N30.01, N39.0 |
| Biliary tract infection | 576.1, 575.0, 574.00 | K83.0, K81.0, K80.00 |
| Brain abscess | 324 | G06.0, G06.1, G06.2 |
| Liver abscess | 572.0 | K75.0 |
| Perianal abscess | 566 | K61 |
| Bacterial meningitis | 320 | G00 |
| Septic arthritis | 711 | M00-M03 |
| Infection of catheter, device, implant, and graft | 996.6, 999.3 | T85.7, T80.2 |
| Peritoneal and retroperitoneal infection | 567 | K65, K67, K68 |
| Osteomyelitis | 730.3, 730.8, 730.9 | M86.9, M90.8 |
| Infective endocarditis | 421 | I33 |

**Table S2.** Baseline Characteristics Patients *after* Propensity-Score Weighting

|  | Non-users  (n=6293) | Febuxostat users  (n=3155) | Allopurinol users  (n=2513) | ASMD |
| --- | --- | --- | --- | --- |
| **Age, year, mean (SD)** | 70 (13) | 70 (12) | 70 (12) | 0.0235 |
| **Male, no. (%)** | 3605 (57.28) | 1853 (58.72) | 1486 (59.12) | 0.0372 |
| **Place of residence, no. (%)** |  |  |  | 0.1428 |
| Urban | 3336 (53.00) | 1679 (53.22) | 1336 (53.16) |  |
| Suburban | 2112 (33.57) | 1062 (33.65) | 836 (33.26) |  |
| Rural | 828 (13.16) | 407 (12.89) | 334 (13.30) |  |
| Unknown | 17 (0.27) | 8 (0.24) | 7 (0.28) |  |
| **Income levels, no. (%)** |  |  |  | 0.0169 |
| Quintile 1 (Lowest) | 1580 (25.12) | 803 (25.45) | 628 (25.00) |  |
| Quintile 2 | 1216 (19.32) | 547 (17.35) | 492 (19.57) |  |
| Quintile 3 | 1503 (23.89) | 787 (24.95) | 589 (23.42) |  |
| Quintile 4 | 743 (11.81) | 377 (11.95) | 298 (11.87) |  |
| Quintile 5 (Highest) | 1250 (19.86) | 640 (20.28) | 506 (20.12) |  |
| Unknown | 1 (0.02) | 1 (0.03) | -- |  |
| **Occupation, no. (%)** |  |  |  | 0.0533 |
| Dependents of the insured individuals | 2474 (39.31) | 1264 (40.06) | 1004 (39.96) |  |
| Civil servants, teachers, military personnel and veterans | 573 (9.11) | 290 (9.19) | 223 (8.85) |  |
| Non-manual workers and professionals | 459 (7.90) | 240 (7.62) | 190 (7.56) |  |
| Manual workers | 2117 (33.64) | 1014 (32.14) | 837 (33.32) |  |
| Other | 670 (10.65) | 347 (10.99) | 259 (10.32) |  |
| **Comorbidities, no. (%)** |  |  |  |  |
| Diabetes mellitus | 4119 (65.45) | 2062 (65.37) | 1615 (64.28) | 0.0246 |
| Hypertension | 6036 (95.92) | 3025 (95.90) | 2415 (96.09) | 0.0096 |
| Dyslipidemia | 3721 (59.13) | 1894 (60.02) | 1473 (58.60) | 0.0289 |
| Liver cirrhosis | 276 (4.38) | 145 (4.60) | 110 (4.37) | 0.0110 |
| SLE | 53 (0.84) | 26 (0.82) | 12 (0.47) | 0.0457 |
| Atrial fibrillation | 387 (6.14) | 188 (5.97) | 157 (6.25) | 0.0117 |
| Peripheral arterial disease | 397 (6.31) | 193 (6.12) | 135 (5.39) | 0.0390 |
| **Medications use, no. (%)** |  |  |  |  |
| ACEi | 395 (6.28) | 181 (5.74) | 152 (6.06) | 0.0227 |
| ARB | 2589 (41.13) | 1307 (41.42) | 1053 (41.88) | 0.0152 |
| CCB | 4783 (76.00) | 2423 (76.81) | 1934 (76.97) | 0.0227 |
| Beta-blockers | 2100 (33.36) | 1040 (32.98) | 852 (33.92) | 0.0199 |
| Diuretics | 3903 (62.02) | 1954 (61.95) | 1565 (62.26) | 0.0063 |
| Aspirin | 1412 (22.44) | 693 (21.96) | 578 (23.00) | 0.0249 |
| Other NSAIDs | 1831 (29.10) | 919 (29.14) | 743 (29.55) | 0.0099 |
| Fibrates | 269 (4.27) | 141 (4.47) | 113 (4.50) | 0.0113 |
| Statin | 2132 (33.88) | 1090 (34.54) | 875 (34.81) | 0.0197 |
| Thiazolidinedione | 131 (2.09) | 62 (1.97) | 49 (1.97) | 0.0086 |

ACEi, angiotensin converting enzyme inhibitor; ARB, angiotensin II receptor blocker; ASMD, absolute standardized mean difference; CCB, calcium channel blocker; NSAIDs, nonsteroidal anti-inflammatory drugs; SLE, systemic lupus erythematosus
